# Identifying and Reducing Stigmatizing Language in Home Health Care With a Natural Language Processing–Based System (ENGAGE): Protocol for a Mixed Methods Study

**DOI:** 10.2196/69753

**Published:** 2025-09-25

**Authors:** Zhihong Zhang, Pallavi Gupta, Stephanie Potts-Thompson, Laura Prescott, Morgan Morrison, Scott Sittig, Margaret V McDonald, Chase Raymond, Jacquelyn Y Taylor, Maxim Topaz

**Affiliations:** 1 Columbia University Data Science Institute New York, NY United States; 2 Columbia University School of Nursing New York, NY United States; 3 Columbia University School of Nursing Center for Research on People of Color New York, NY United States; 4 University of Louisiana at Lafayette Health Sciences Lafayette, LA United States; 5 Center for Home Care Policy & Research VNS Health New York, NY United States; 6 Department of Linguistics University of Colorado Boulder, CO United States; 7 Department of Family Medicine Anschutz Medical Campus University of Colorado Aurora, CO United States

**Keywords:** natural language processing, racial bias, stigmatizing language, home health care, artificial intelligence, AI

## Abstract

**Background:**

Stigmatizing language is common in clinical notes and can adversely affect the quality of patient care. Natural language processing (NLP) is a promising technology for identifying such language across large volumes of clinical notes in electronic health records.

**Objective:**

This study proposes an NLP-driven reduce stigmatizing language (ENGAGE) system to automatically identify and replace stigmatizing language.

**Methods:**

Using a mixed methods study, we will extract electronic health record data for patients admitted to 2 large, diverse home health care (HHC) organizations between January 2019 and December 2021. We propose the following 4 aims: aim 1 is to refine the ontology of stigmatizing language in HHC by (1) interviewing a diverse sample of HHC nurses and patients to identify terms to avoid and (2) analyzing clinical notes from various regions in the United States to categorize stigmatizing language. Aim 2 is to determine the best NLP approach for accurately identifying stigmatizing language by training algorithms and comparing their performance to human annotations. Aim 3 is to analyze the prevalence of stigmatizing language based on patients’ race and ethnicity using adjusted statistical analyses of a sample of approximately half a million HHC patients (34% racial and ethnic minority groups). Aim 4 is to develop the NLP-driven ENGAGE system by (1) testing NLP methods (rule based; “delete, retrieve, and generate”; and transformers) for suggesting alternative wording and (2) designing and refining the user interface for clinical trial preparation.

**Results:**

We received funding from the National Institute on Minority Health and Health Disparities in September 2023. Recruitment began in May 2024, and as of March 2025, interviews have been completed for 9 enrolled participants. We anticipate completing all study aims by April 2027.

**Conclusions:**

This study will leverage extensive data sources to examine stigmatizing language in HHC settings and contribute to the development of systems aimed at effectively reducing the use of such language among HHC nurses.

**International Registered Report Identifier (IRRID):**

DERR1-10.2196/69753

## Introduction

Home health care (HHC) is one of the fastest-growing outpatient settings in the United States, where 200,000 nurses provide care for more than 5 million patients annually [1,2]. Although the quality of nursing care is affected by numerous factors (eg, structural resources, levels of education, and patient-per-nurse ratios) [3-5], nurses’ biases toward their patients influence the delivery of high-quality care [6]. Implicit bias is the negative inclination of one group and its members relative to others unconsciously or unintentionally [6]. Recent literature reviews [7,8] found widespread, implicit biases among nurses toward their patients. Specifically, a recent review of 215 studies [9] found that nurses frequently display biases based on patients’ race or ethnicity, influencing treatment decisions and impacting patient adherence and outcomes [10,11].

Racial biases can be propagated via the language used in electronic health record (EHR) documentation [12-15]. Research has shown that stigmatizing language used in clinical notes can harm patient care [12-15]. Patients for whom stigmatizing language was used had HHC visits that were 24 minutes shorter than those whose records were without such language (average visit length 46 vs 70 minutes, respectively) [16,17]. This is concerning because shorter HHC visits are associated with poor outcomes (eg, higher risk of hospitalizations) [10-12,16-22]. A recent study found that 10% of 22,959 patients who reviewed their clinical notes felt judged or offended by stigmatizing language [23]. This is crucial because, as of April 2021, health care organizations, including HHC, must share EHR data with patients under the 21st Century Cures Act’s “Information Blocking” Rule [24]. More than 80% of HHC agencies have EHRs, and reducing stigmatizing language use can decrease racial biases and improve the quality of care and patient outcomes [25].

When applied appropriately, technology can help identify and reduce biases in health care [26]. One promising technology is natural language processing (NLP), a branch of artificial intelligence that analyzes free-text data, such as clinical notes in EHRs, to extract meaningful insights [27]. NLP has been used to detect health care provider biases by identifying stigmatizing language in clinical notes [28]. Common NLP techniques include rule-based methods such as NimbleMiner [29], which identify specific stigmatizing terms [30,31], as well as machine learning and deep learning models such as the clinical bidirectional encoder representations from transformers (BERT) model, which can detect more subtle, context-dependent patterns in the language [32]. In our previous research, we applied NimbleMiner to identify stigmatizing language in clinical notes and found that 38% of notes contained such language. In addition, notes about Black patients had up to 50% higher odds of containing stigmatizing language than those about White patients [30].

Given the high prevalence of stigmatizing language, what it represents in relation to bias, and its negative impact on patient care, this study proposes an NLP-driven reduce stigmatizing language (ENGAGE) system to automatically identify and address bias and replace stigmatizing language in clinical notes.

## Methods

### Ethical Considerations

This research was approved by Columbia University’s institutional review board (AAAU7957). Eligible and willing participants will provide informed consent via Qualtrics (Qualtrics International Inc) or verbal consent (patient only). Participants can choose to opt out at any point throughout the study. All data will be deidentified to protect participant privacy. The participants will be compensated US $50 in the form of an Amazon electronic gift card for a one-time interview. Nurses serving as content experts will return for a second interview and will receive a US $100 Amazon electronic gift card. Compensation transparency is ensured through recruitment flyers and consent forms, both of which contain the compensation type and amount.

### Study Design

**Figure 1:**
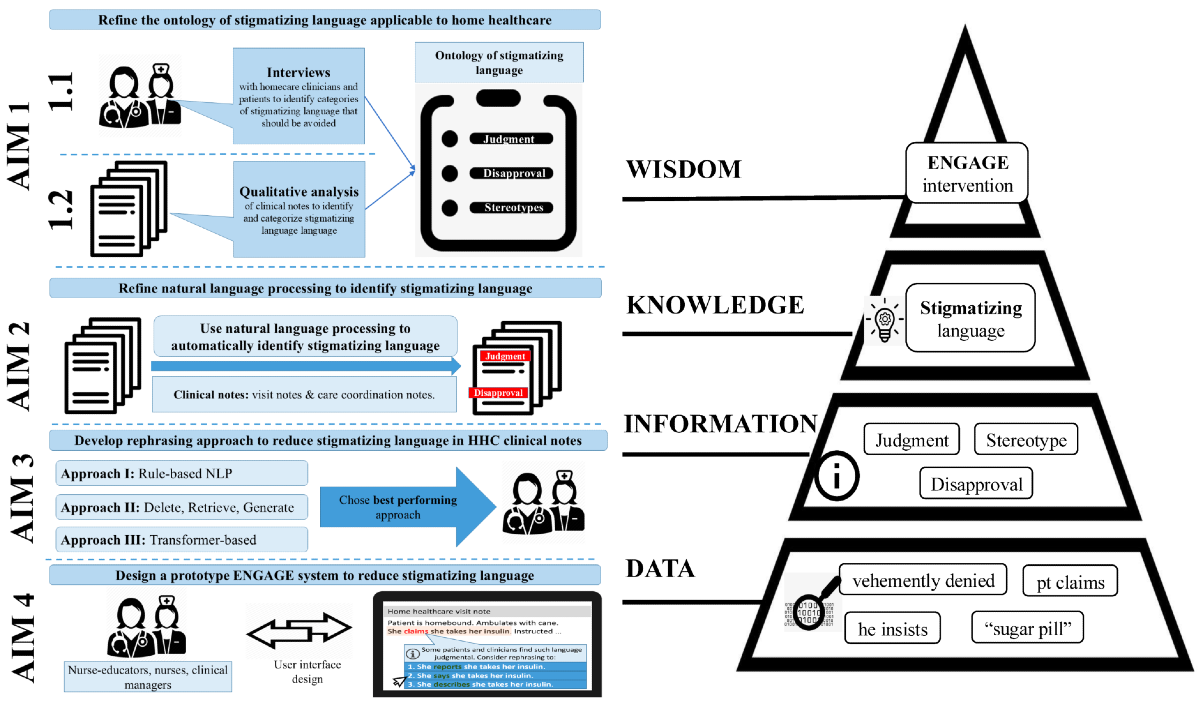
Reduce stigmatizing language (ENGAGE) study design. HHC: home health care; NLP: natural language processing.

We proposed 4 corresponding study aims that followed a mixed methods study design to achieve study goals (Figure 1). This protocol is reported in accordance with the Standards for Reporting Implementation Studies statement [33], except for the Results section, which is reported according to *JMIR*’s requirements for protocols. In aim 1, we will adapt the ontology of stigmatizing language for HHC via interviews with patients and nurses and qualitative analysis of clinical notes. In aim 2, we will develop and compare several NLP approaches to automatically identify stigmatizing language in clinical notes. In aim 3, we will compare the prevalence of stigmatizing language by patients’ race and ethnicity. In aim 4, we will develop an NLP-driven ENGAGE system to reduce the use of stigmatizing language in clinical notes. This 4-aim study design is informed by the data, information, knowledge, and wisdom conceptual framework [34,35]. This framework suggests that discrete data points generate meaningful information that can be turned into knowledge. Wisdom is the appropriate use of knowledge to manage and solve problems. We will identify stigmatizing language from interviews and clinical notes (data: aims 1 and 2), categorize it (information: aims 1 and 2), analyze its associations (knowledge: aim 3), and apply the findings to develop the intervention (wisdom: aim 4; Figure 1). This study aligns with the National Institute on Minority Health and Health Disparities’ framework, focusing on health care and interpersonal and individual levels in HHC nursing [36].

### Study Setting and Data Sources

This study will be conducted within 2 diverse, large HHC organizations: one is a large not-for-profit HHC agency serving patients in New York City and its surrounding suburbs. The other is a national HHC provider network with more than 300 HHC agencies in more than 30 states in the United States. EHR data will be extracted for patients admitted to HHC between January 1, 2019, and December 31, 2021 (3 years). We expect to include approximately half a million unique patients. The expected patient demographics will be 67% White (non-Hispanic), 15% Black (non-Hispanic), 11% Hispanic, 2.5% Asian, and 4.5% other (including American Indian, Alaska Native, Native Hawaiian, and Pacific Islander). This should yield over 16 million clinical notes, accounting for the involvement of over 10,000 HHC nurses.

The following study variables will be extracted from the EHR (Table 1): (1) the Outcome and Assessment Information Set (OASIS)—OASIS is a comprehensive, Centers for Medicare & Medicaid Services–mandated standardized assessment tool designed to collect nearly 100 items related to a recipient’s functional status, clinical status, and service needs during an HHC episode [37]—(2) administrative data: human resources data will be used to extract HHC nurse characteristics; and (3) clinical notes: the stigmatizing languages and their classifications will be identified at the clinical notes level in aim 2.

**Table 1:** Study variables and sources.

Variable categories	Variables	Data source
Sociodemographics	Age at start of care, race, ethnicity, sex, and geographic location	OASIS^a^
Physiological measures	Height, weight, and BMI	OASIS
Functional status	Activities of daily living and disability	OASIS
Cognitive status	Cognitive impairment	OASIS
Clinical information	Diagnosis and comorbid conditions	OASIS
Presence of stigmatizing language	Categories of stigmatizing language extracted via NLP^b^	Clinical notes

^a^OASIS: Outcome and Assessment Information Set.

^b^NLP: natural language processing.

### Participant Interviews and Recruitment

We will conduct interviews with a sufficient maximum variation sample of 35 HHC nurses and 35 patients in aim 1 [38]. For nurses, we aim to create a diverse sample stratified by race and ethnicity, years of experience (<5 years vs ≥5 years), and geographic location [39-41]. HHC nurses will be enrolled if they are currently being employed by the participating HHC organizations. About 15 (43%) of those nurses will serve as content experts and partake in an additional interview in aim 4. For patients, we will get a sufficient maximum variation sample stratified by race and ethnicity, sex, and geographic location. Patients will be included if they are aged ≥18 years and recently admitted to or discharged from HHC within the last 3 years.

Nurses will be recruited through email advertisements and presentations at nursing team meetings. Patients will be recruited through direct outreach (ie, phone calls) based on records of recently treated and discharged patients.

### Study Procedures

#### Aim 1: Identify the Ontology of Stigmatizing Language

On the basis of our previous work [29,42-44], the ontology of stigmatizing language will be refined and expanded from the interviews with HHC patients and nurses and a review of clinical notes.

#### Participant Interview

To generate a sufficient maximum variation sample, we plan on conducting about 30 to 35 interviews for HHC nurses and patients separately. Interviews will continue until data saturation is reached, lasting up to 2 hours and conducted by phone, Zoom (Zoom Video Communications, Inc), or in person (for patients only). An interview guide with semistructured and open-ended questions will be used. These guides were developed to facilitate discussions with nurses (Multimedia Appendix 1) and patients (Multimedia Appendix 2), aiming to refine the categories of stigmatizing language to avoid. Example questions include the following: “Have you noticed negative, discriminatory, or stigmatizing language used by HHC clinicians? Please explain.” “We found expressions like ‘claims smoking cessation, but ashtray still noted on nightstand’ in the HHC clinical notes. Would you consider this judgmental or offensive? Should this language be changed or eliminated?” All interviews will be audio-recorded for analysis.

#### Clinical Notes

A subset of clinical note samples will be selected to identify stigma language in clinical notes. We will aim for a maximum variation sample of clinical notes. This sample will be stratified by (1) geographically diverse HHC agencies (Northeastern, Midwestern, and Southern United States and the New York City boroughs); (2) urban, suburban, and rural areas; (3) diverse patient populations; and (4) HHC nurses of varying sex, race, and ethnicity, years of experience, educational levels, and geographic locations. On the basis of our pilot work [29,42-44], we estimate that 10% to 20% of clinical notes will contain stigmatizing language. To capture linguistic patterns fully, we will initially analyze 10,000 clinical notes, with additional batches of 2500 notes if necessary to reach knowledge saturation [45]. We will annotate each clinical note for stigmatizing language and its categories (eg, “Stereotyping by race or social class” or “Portraying the patient as difficult”) [13]. Using a hybrid, qualitative approach of inductive and deductive coding [46], we will begin with the 5 categories from our previous study and refine or add categories as needed through discussions with annotators, the study team, and the Stakeholders Engagement Board (SEB; an interdisciplinary team of experts). Four annotators will review each note: 2 experienced HHC nurses (1 White and 1 from a minority group), a social worker with racial bias detection expertise, and a minority patient who received HHC services. Annotations will be done using Amazon Web Services SageMaker Ground Truth [47], with each annotator independently marking instances of stigmatizing language. Results will be merged and reviewed. Interrater reliability will be tracked using κ statistics, aiming for strong agreement (>0.8) [48].

#### Aim 2: Determine the Optimal NLP Approach for Stigmatizing Language Identification

We will evaluate and compare 3 NLP approaches using SageMaker Ground Truth. The first approach, key-term discovery with NimbleMiner [29,43,49,50], will build on previous work by creating vocabularies of synonyms and excluding irrelevant terms, followed by machine learning classification using models such as XGBoost, random forest, support vector machines, and long short-term memory neural networks, with predictions reviewed until saturation. The second approach involves fine-tuning a publicly available clinical BERT model [51,52] trained on a large set of clinical notes [53] using our HHC notes to improve language representation. The third NLP approach includes 2 aims: feature generation and model training. Feature generation will use techniques such as one-hot encoding; term frequency–inverse document frequency; word embedding techniques (ie, Skip-Gram [54], Glove [55], and FastText [56]); and dynamic (conceptualized) word embedding techniques (ie, ELMO [57] and clinical BERT [52]). During model training, machine learning classifiers will be trained and validated in the model training to identify stigmatizing language in clinical notes. To enable this comparison, the annotated sample of approximately 10,000 clinical notes from aim 1 will be split into training (60%), validation (10%), and testing (30%) sets, stratified by stigmatizing language categories.

#### Aim 3: Compare the Prevalence of Stigmatizing Language by Patients’ Race and Ethnicity

On the basis of our pilot work in hospital and ambulatory settings and HHC, we hypothesize that 2 or more stigmatizing language categories (eg, questioning patient credibility [ie, judgment]; stereotyping by race or social class) will be associated with the patient’s race and ethnicity. We define race and ethnicity based on the categories available in the federally mandated HHC assessment data (OASIS) [36] that we will use in the study, as follows: non-Hispanic Black, Hispanic, Asian or Pacific Islander, American Indian or Alaska Native, and non-Hispanic White. The data available in federally mandated OASIS are 99% complete (ie, no missing data) based on our 2 decades of experience working with these data. Potential covariates will be identified from the data resources of OASIS and administrative data.

#### Aim 4: Develop an NLP-Driven ENGAGE System

To enable the development of an NLP-driven ENGAGE system, we will first identify the best method for rephrasing stigmatizing language without altering meaning. Three approaches will be compared: a rule-based method using synonym lists reviewed by annotators; a “delete, retrieve, and generate” method that modifies stigmatizing attributes [58]; and transformer-based models (eg, BERT [51], generative pretrained transformers 3 [59], and text-to-text transfer transformer [60]). Two datasets will be created and used for this comparison, including a training set of 2500 clinical notes with stigmatizing language (500 examples per category) and a test set of 4000 sentences. In total, 5 reviewers will rewrite the sentences and reach a consensus through Delphi rounds, generating sentence pairs for training and testing. For this task, we define NLP performance as (1) the system’s ability to replace stigmatizing language with nonstigmatizing neutral language and (2) the system’s ability not to alter the meaning of the source sentence significantly. The identified NLP approach with the best performance will help paraphrase stigmatizing language without significantly changing the original text’s meaning in the NLP-driven ENGAGE system.

**Figure 2:**
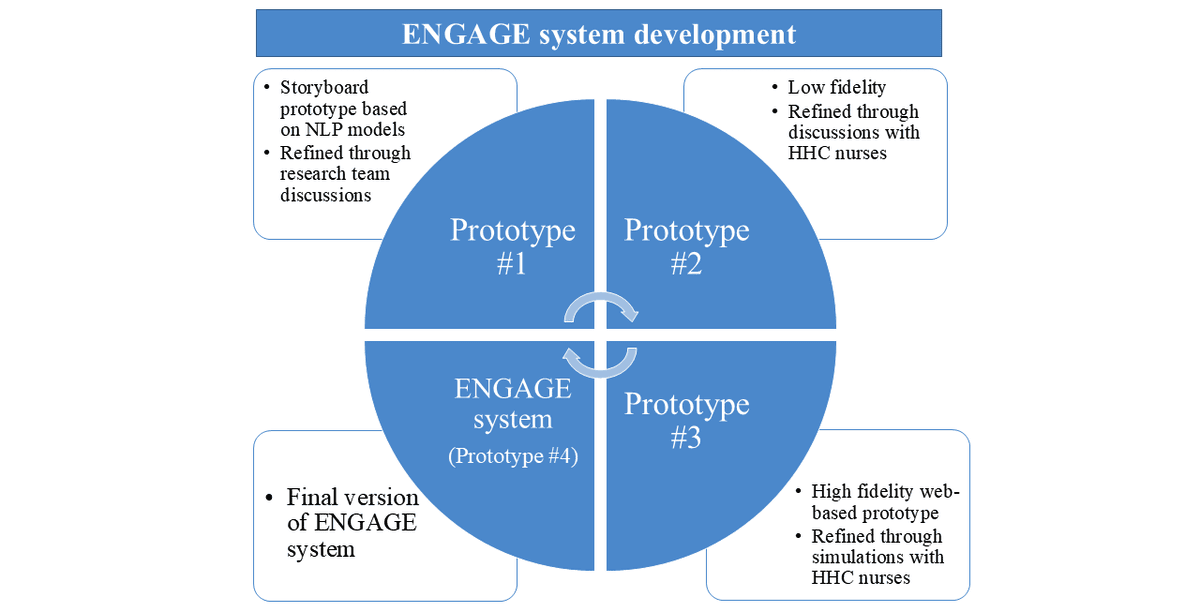
Iterative development process of the reduce stigmatizing language (ENGAGE) system. HHC: home health care; NLP: natural language processing.

**Figure 3:**
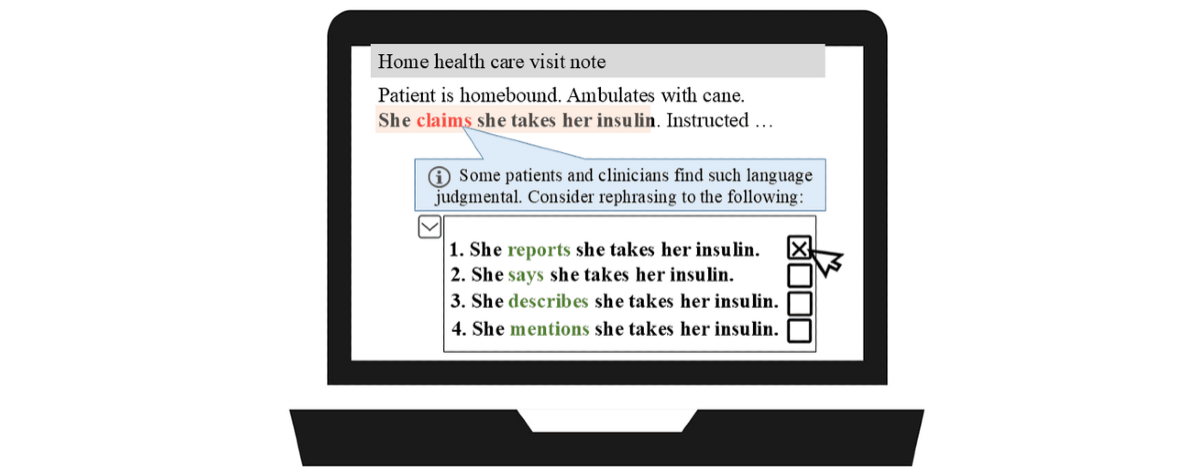
User interface of a potential stigmatizing language reduction system.

Next, the best NLP approach will be incorporated into the NLP-driven ENGAGE system. Iterative user-centered design methodologies will be used (Figure 2) to develop the NLP-driven ENGAGE system based on agile software development approaches [61,62] that were implemented by our research team in numerous previous studies [63-67]. We will start with an initial storyboard prototype (prototype 1) and refine it through team discussions, leading to several low-fidelity prototypes (prototype 2). These will be reviewed with a subset of HHC nurses from aim 1 and SEB, resulting in a high-fidelity web-based prototype (prototype 3) built with the Shiny visualization package in R (R Foundation for Statistical Computing). This iterative process will continue until a final user interface (Figure 3) is developed, addressing key questions about screen layout; visualization of recommendations; delivery methods (pop-up, dashboard icon, and message); and timing within clinician workflows.

### Data Analysis Plan

In aim 1, interview audio data will be transcribed by the research assistant, with 20% to 30% validated by another study team member. Data will be analyzed using thematic analysis—a qualitative descriptive approach for identifying, analyzing, and reporting themes within the data [68-74]. Qualitative analysis software (NVivo [75]; Lumivero) will be used to implement the analysis. The analysis includes six aims: (1) familiarization with data by listening to recordings and reading transcripts; (2) generating initial codes based on interview questions; (3) data coding by 2 researchers with dual coding to ensure >90% agreement; (4) collating codes into themes; (5) defining and naming themes; and (6) producing a final report with quotes, linking themes to the research question and the literature.

In aim 2, the performance of 3 NLP approaches will be compared on the testing set to identify the best one for identifying stigmatizing language. For each stigmatizing language category, we will calculate the area under the receiver operating characteristic curve (AUC-ROC), area under the curve of precision recall (AUC-RP), and the *F* score (a harmonic mean between precision and recall). AUC-ROC is the tradeoff between a true positive rate (sensitivity) and a false positive rate (1–specificity). It has the advantage of being invariant to the class distribution but does not provide sufficient information about the model’s precision [76]. On the other hand, AUC-RP is the tradeoff between recall (true positive rate) and precision [76]. Because our goal is to maximize the sensitivity and precision of the NLP systems in identifying clinical notes with stigmatizing language, we will rank the performance of NLP systems using AUC-RP. We aim to achieve an *F* score and AUC-ROC >0.80, which indicates a well-balanced and functioning system. If NLP approaches fail to achieve this performance level, we will conduct another cycle or cycles of data annotation (with increments of 2500 clinical notes) and NLP system fine-tuning until the desired performance is achieved.

In aim 3, we will compare the prevalence of stigmatizing language by patients’ race and ethnicity. The dependent variable will be the presence of stigmatizing language in the clinical note (yes and no). Analyses will be conducted at the clinical note level, starting with bivariate assessments of potential confounders (eg, sex, age, disease diagnosis, and comorbidities). Significant variables (*P*<.05) will be included in mixed-effects regression models to examine associations between race and ethnicity and stigmatizing language, accounting for clustering within patients and nurses. If stigmatizing language is infrequent, mixed-effects Poisson or negative binomial regression will be used. We will control the false discovery rate at 0.05 for multiple comparisons.

In aim 4, we will evaluate the best approach for rephrasing stigmatizing language. Each NLP method will generate 4 paraphrased options from the test set. Five human reviewers will independently select options that replace stigmatizing language without altering the sentence’s meaning. The research group will then conduct Delphi rounds to reach a consensus on the best versions. A new group of 5 reviewers, including diverse HHC nurses, a minority patient, and an SEB chair, will rate each rephrased sentence on a 5-point Likert scale for effectively replacing stigmatizing language and preserving meaning. This group will first independently review each rephrased sentence and, on a 5-point Likert scale (range—1=almost completely, 2=to some extent, 3=unsure, 4=to a small extent, and 5=did not change), indicate (1) to what extent stigmatizing language was replaced with a neutral, nonstigmatizing language and (2) to what extent the meaning of the source sentence was altered. We will generate mean and median scores for each NLP approach and examine whether any of the NLP approaches achieved statistically significantly better performance on questions 1 (replace stigmatizing language) and 2 (not changing the meaning of the sentence) using ANOVA [77].

## Results

We received funding from the National Institute on Minority Health and Health Disparities on September 24, 2023, with a project end date of April 30, 2027. Recruitment and enrollment began in May 2024. As of August 2025, we enrolled 13 participants. Table 2 presents the planned timeline and current progress for each study phase.

**Table 2:** Timeline and study progress.

Phase	Planned timeline	Current progress
Aim 1	May 2024-December 2025	Interviews completed for 9 participants
Aim 2	January 2025-December 2025	Data processed for analysis
Aim 3	January 2026-April 2026	Not yet initiated
Aim 4	May 2026-April 2027	Not yet initiated

## Discussion

### Anticipated Findings

Reducing racial biases in health care is a national priority. This innovative study will leverage extensive data sources to explore stigmatizing language in clinical notes, addressing critical gaps in detecting racial bias in EHRs and improving system design to minimize such language used by HHC nurses. The research team includes qualified researchers who will ensure the study’s implementation and timely completion.

One expected outcome of this study is the identification of an expanded ontology of stigmatizing language categories. In a previous study, 5 categories were identified: questioning patient credibility, expressing disapproval of patient reasoning or self-care, stereotyping by race or social class, portraying the patient as “difficult,” and emphasizing clinician authority over the patient [13]. However, these categories were derived from 600 encounter notes written by 138 physicians. This study will expand the data to a larger sample of clinical notes and interviews with patients and nurses. With this larger dataset and diverse perspectives, the expectation is to identify additional categories of stigmatizing language. These expanded and refined categories will allow for a more comprehensive analysis of stigmatizing language in clinical notes.

The prevalence of stigmatizing language is expected to vary across different racial and ethnic groups. Using data from one of the largest not-for-profit HHC agencies in the United States, a pilot NLP study was conducted to examine stigmatizing language use among HHC nurses. The study found that stigmatizing language was least prevalent in the Asian group of patients. Compared to this group, the prevalence increased by 22%, 37%, and 39% in the White, Black, and Hispanic groups, respectively [12]. In this study, using a similar population, it is expected that these racial and ethnic differences will persist. Therefore, it is crucial to develop interventions to reduce racial bias and stigmatizing language in clinical notes.

Several NLP approaches, such as rule-based methods and BERT-based models, have been evaluated for identifying stigmatizing language in clinical notes [15,28,31,78,79]. These approaches have their limitations. For example, rule-based approaches, which rely on predefined vocabularies, are rigid and often miss context-dependent nuances, while transformer-based models such as clinical BERT capture context better but are limited by the training data. Recent advancements in large language models, such as Mistral and Large Language Model Meta AI 3 [80,81], have improved the performance across various NLP benchmarks. Therefore, future research can explore and compare these newer models in addressing stigmatizing language in clinical notes.

### Strengths and Limitations

This study has several strengths. First, this is an original study that will examine the ontology of stigmatizing language and explore the NLP approach to automatically identify and reduce stigmatizing language use in HHC. Second, the strengths of our study include the rich data resource of approximately 16.7 million clinical notes for about 667,000 unique HHC patients. This rich dataset enables a comprehensive understanding of racial bias in the language used in clinical notes. Third, an interdisciplinary team of experts in linguistics, health disparities, HHC nursing, qualitative analysis, and NLP has been assembled to design a nurse-centered, NLP-based system. With strong expertise in both content and methodology, the team has carefully considered potential biases and limitations, developing a plan to address them and enhance scientific rigor.

Limitations have also been identified. First, stigmatizing language can be ambiguous and difficult to identify. To mitigate this, the interdisciplinary team of experts includes experts in racial health disparities. In addition, data annotators will represent diverse racial perspectives. Various “interrater reliability” steps will be included within the protocol to reduce the potential for such ambiguities and others that may be overlooked. Second, developing an effective ENGAGE system will pose challenges. To address this, a comprehensive, iterative development plan will be created with input from diverse HHC clinicians.

### Conclusions

The ENGAGE study protocol addresses the critical issue of stigmatizing language in HHC through developing an NLP-driven system. This innovative mixed methods study, conducted within 2 large and diverse HHC organizations, aims to refine the ontology of stigmatizing language, develop and test various NLP methods for identifying and replacing such language, and examine the prevalence and impact of stigmatizing language across different racial and ethnic groups. The project will comprehensively analyze the patterns and consequences of stigmatizing language in clinical notes by leveraging extensive EHR data and using robust statistical and machine learning techniques. With the successful development and iterative refinement of the NLP-driven ENGAGE system, which integrates the most effective NLP approach for rephrasing stigmatizing language without altering the original meaning, the final system will be ready for testing in a clinical trial.

## Appendix

Multimedia Appendix 1 Interview guide for nurses.

Multimedia Appendix 2 Interview guides for patients.

## Abbreviations

AUC-ROC: area under the receiver operating characteristic curve
AUC-RP: area under the curve of precision recall
BERT: bidirectional encoder representations from transformers
EHR: electronic health record
ENGAGE: reduce stigmatizing language
HHC: home health care
NLP: natural language processing
OASIS: Outcome and Assessment Information Set
SEB: Stakeholders Engagement Board

## Data Availability

The datasets generated or analyzed during this study are available from the corresponding author upon reasonable request.
