# Supplementary material for: Identifying and Reducing Stigmatizing Language in Home Health Care With a Natural Language Processing–Based System (ENGAGE): Protocol for a Mixed Methods Study
Source: JMIR Res Protoc. 2025 Sep 25;14:e69753. doi: 10.2196/69753 (PMC12511817; doi:10.2196/69753)
Supplement: Multimedia Appendix 1 [file resprot_v14i1e69753_app1.docx]

**Interview Guide for Homecare Nurses**

**Introduction**: Thank you for participating in this interview. Your insights are vital in identifying and understanding judgmental, offensive, or stigmatizing language within HHC. Your confidentiality will be maintained. If at any time you have a question or need clarification, please let me know.

**Section 1: Awareness and Perception of Judgmental, Offensive, or Stigmatizing Language**

- Are you familiar with instances of judgmental, offensive, or stigmatizing language in HHC clinical notes? Can you provide examples or reactions to those?
- How does this type of language affect patient care and professional relationships?

**Section 2: Assessment of Specific Examples**

- How do you perceive the use of the word "claims" in notes such as "...claims smoking cessation but ashtray still noted on nightstand" or "pt claims he had fever in past, but no thermometer in use"? Is this language judgmental, offensive, or stigmatizing? How should it be changed?
- What are your thoughts on the use of "insisted" or "insists" as in "He has a rollator walker, but patient only uses it to get up from the bed, but patient insisted on doing it his way" or "patient also insisted visiting nurse to remove left foot dressing however no wound order suggested to do so"? How would you classify this language?
- How do you perceive the use of "adamant" or "adamantly" in phrases like "has a rollator walker, but husband is so adamant for patient not to use it" or "Patient has a straight cane but adamantly refused it in the appointment and patient prefer holding on walls and furniture"? Is this language judgmental, offensive, or stigmatizing?
- What is your perception of the terms "states" and "admits" in contexts such as "Patient says she feels weak and dizzy patient admits to not testing blood sugars as ordered but states she takes her insulin" or "Patient admits to not testing blood sugars as ordered but states she takes her insulin"? How does this language appear to you?
- How do you view the use of "convinced" and "claims" in notes like "patient refuses to wash legs and claims he is allergic to water”, “patient convinced generic medicine is only solution for his wound care treatment" or "patient has diabetes mellitus (DM) and heart failure (HF), but convinced they don't need to keep low sugar diet"? Do you find this language to be judgmental, offensive, or stigmatizing?

**Section 3: Identification of Additional Judgmental, Offensive, or Stigmatizing Language**

- Can you identify any additional terms or expressions that may be considered judgmental, offensive, or stigmatizing? Please explain why these are considered problematic and provide suggestions for alternative language.

**Section 4: Impact on Clinical Practice and Suggestions for Improvement**

- How do these examples of language influence care quality, patient-provider relationships, and integrity within HHC?
- What strategies or guidelines would you recommend to minimize or eliminate the use of judgmental, offensive, or stigmatizing language in HHC?

**Conclusion**: Your input is crucial in refining our understanding of judgmental, offensive, or stigmatizing language in HHC and finding ways to enhance clinical practice. Thank you for your time and insights.
