# Supplementary material for: Identifying and Reducing Stigmatizing Language in Home Health Care With a Natural Language Processing–Based System (ENGAGE): Protocol for a Mixed Methods Study
Source: JMIR Res Protoc. 2025 Sep 25;14:e69753. doi: 10.2196/69753 (PMC12511817; doi:10.2196/69753)
Supplement: Multimedia Appendix 2 [file resprot_v14i1e69753_app2.docx]

**Interview Guide for Homecare Patients**

**Introduction**: Thank you for agreeing to take part in this interview. We value your perspective as a patient in understanding the use of judgmental, offensive, or stigmatizing language in home healthcare. Your identity and responses will be kept confidential. If at any time you have a question or need clarification, please let me know.

**Section 1: Awareness and Impact of Judgmental, Offensive, or Stigmatizing Language**

- Have you ever come across or felt that the language used in HHC notes or communication with nurses was judgmental, offensive, or stigmatizing? Could you describe the situation?
- How has this type of language affected your patient experience, relationship with healthcare providers, or perception of care?

**Section 2: Reactions to Specific Examples**

- How do you feel about the use of the term "claims" in sentences like "...claims smoking cessation but ashtray still noted on the nightstand"? Do you find this language judgmental, offensive, or stigmatizing?
- What are your thoughts on the word "insisted" in phrases such as "He has a walker but the patient only uses it to get up from the bed, but the patient insisted on doing it his way"? Does this language resonate with you in any particular way?
- How do you react to the use of "adamant" in contexts like "has a walker, but the husband is so adamant for patient not to use it"? What are your feelings about this language?
- How do the terms "states" and "admits" in examples like "Patient says she feels weak and dizzy patient admits to not testing blood sugars as ordered but states she takes her insulin" make you feel? Are these terms judgmental, offensive, or stigmatizing to you?
- What is your perception of the use of "convinced" and "claims" in notes such as "patient refuses to wash legs and claims he is allergic to water” or “patient convinced generic medicine is the only solution for his wound care treatment"? How does this language make you feel?

**Section 3: Identification of Additional Concerning Language**

- Can you identify any other words or phrases in home healthcare communication that have made you feel uncomfortable, judged, or stigmatized? Could you provide examples and explain why they are problematic?

**Section 4: Suggestions for Improving Communication in HHC**

- What changes would you like to see in the way healthcare providers communicate with you or document care? Are there specific terms or approaches that you believe would create a more respectful and compassionate environment?

**Conclusion**: Your insights are instrumental in helping us understand the impact of judgmental, offensive, or stigmatizing language in HHC. We appreciate your time and willingness to share your experiences.
